# Blood Microbiota Dysbiosis Is Associated with the Onset of Cardiovascular Events in a Large General Population: The D.E.S.I.R. Study

**DOI:** 10.1371/journal.pone.0054461

**Published:** 2013-01-25

**Authors:** Jacques Amar, Céline Lange, Gaëlle Payros, Celine Garret, Chantal Chabo, Olivier Lantieri, Michael Courtney, Michel Marre, Marie Aline Charles, Beverley Balkau, Rémy Burcelin, D.E.S.I.R. Study Group

**Affiliations:** 1 INSERM U1048, Institute of Research on Metabolic and Cardiovascular Diseases, CHU Toulouse, Toulouse, France; 2 INSERM, CESP Centre for research in Epidemiology and Population Health, U1018, Epidemiology of Diabetes, Obesity and Chronic Kidney Disease Over the Lifecourse, Villejuif, France; 3 University Paris Sud 11, UMRS 1018, Villejuif, France; 4 Bio-Medical Research Federative Institute of Toulouse, Toulouse, France; 5 IRSA, La Riche, France; 6 VAIOMER SAS, Toulouse, France; 7 Service d'endocrinologie-diabète-nutrition, Groupe Hospitalier Bichat-Claude Bernard, Assistance Publique des Hôpitaux de Paris, Paris, France; 8 INSERM U695, Université Denis Diderot Paris 7, Paris, France; Cardiff University, United Kingdom

## Abstract

**Aim:**

We recently described a human blood microbiome and a connection between this microbiome and the onset of diabetes. The aim of the current study was to assess the association between blood microbiota and incident cardiovascular disease.

**Methods and Results:**

D.E.S.I.R. is a longitudinal study with the primary aim of describing the natural history of the metabolic syndrome and its complications. Participants were evaluated at inclusion and at 3-, 6-, and 9-yearly follow-up visits. The 16S ribosomal DNA bacterial gene sequence, that is common to the vast majority of bacteria (Eubac) and a sequence that mostly represents Proteobacteria (Pbac), were measured in blood collected at baseline from 3936 participants. 73 incident cases of acute cardiovascular events, including 30 myocardial infarctions were recorded. Eubac was positively correlated with Pbac (r = 0.59; *P*<0.0001). In those destined to have cardiovascular complications, Eubac was lower (0.14±0.26 vs 0.12±0.29 ng/µl; *P* = 0.02) whereas a non significant increase in Pbac was observed. In multivariate Cox analysis, Eubac was inversely correlated with the onset of cardiovascular complications, (hazards ratio 0.50 95% CI 0.35–0.70) whereas Pbac (1.56, 95%CI 1.12–2.15) was directly correlated.

**Conclusion:**

Pbac and Eubac were shown to be independent markers of the risk of cardiovascular disease. This finding is evidence for the new concept of the role played by blood microbiota dysbiosis on atherothrombotic disease. This concept may help to elucidate the relation between bacteria and cardiovascular disease.

## Introduction

Treatment of traditional cardiovascular risk factors has resulted in a dramatic decrease in the incidence of cardiovascular disease over the past 30 years [1], [2]. Despite progress, cardiovascular disease remains the leading cause of mortality throughout the world, including in developed countries. More importantly, even in well treated and controlled high-risk cardiovascular patients, an important residual cardiovascular risk persists [3], [4]. Beside the role of traditional risk factors, experimental and epidemiological evidence that infection is involved in the onset of atherosclerotic diseases continues to mount [5]–[11].Interestingly, the role of gram negative bacteria on arterial wall structure and function has been studied. Wiederman et al [12] showed an association between endotoxemia and carotid atherosclerosis in a prospective population-based study. In line with this data, our group reported in a population-based study, a correlation between aortic stiffness and soluble CD14 blood level, the main endotoxin receptor [13]. A randomized trial showed that a vaccination against gram negative bacteria resulted in aortic stiffening [14]. Furthermore, the presence of bacteria from intestinal or oral origin have been observed in atherosclerotic plaques and importantly, compared with oral and gut samples, atherosclerotic plaque contained significantly higher levels of Proteobacteria (Pbac) and fewer Firmicutes [15]. In this respect, we recently described a human blood microbiome [16]. As for gut microbiota, this result introduced the concept of tissue microbiota equilibrium as a potential factor in human health. Indeed, a body of evidence shows that gut microbiota dysbiosis plays a key role in various diseases such as type 1 diabetes [17], bowel diseases [18], [19] and celiac disease [20]. For example, in inflammatory bowel diseases, reduced microbial diversity was observed [18]. Fecal samples from patients with celiac disease had lower proportions of *Bifidobacterium* and increased proportions of *Bacteroides*/*Prevotella* [20]. In the light of these data, rather than analyse the role of few selected pathogens, we sought to explore the effect of blood microbiota equilibrium on the onset of cardiovascular diseases in a general population, with a focus on proteobacteria phylum.

## Methods

### Population

D.E.S.I.R. (Data from an Epidemiological Study on the Insulin Resistance syndrome) is a longitudinal cohort study of 5,212 adults aged 30–65 years at baseline; the primary aim of the study was to describe the natural history of the metabolic syndrome [21]. Participants were recruited in 1994–1996 from ten Social Security Health Examination centers in central-western France, from volunteers insured by the French national social security system (80% of the French population - any employed or retired person and their dependents are offered free periodic health examinations). Equal numbers of men and women were recruited in five-year age groups. All participants gave written informed consent, and the study protocol was approved by the CCPPRB (Comité Consultatif de Protection des Personnes pour la Recherche Biomédicale) of the Hôpital Bicêtre (Paris, France). Participants were clinically and biologically evaluated at inclusion and at 3-, 6, and 9-yearly follow-up visits. We excluded participants without data on the Pbac gene, Eubac gene, LDL-cholesterol, smoking, diabetes, hypertension or fibrinogen, and those who were likely to have infections: fibrinogen >5 mg/l, abundant leucocyturia, taking antiviral therapy and extreme blood bacterial DNA levels.

### Parameters Studied

The examining physician recorded the history of cardiovascular diseases and treatment for diabetes and hypertension. Smoking habits were documented in a self-administered questionnaire. Smokers were defined as those currently smoking ≥1 cig/day at inclusion or who quit smoking less than 3 years prior to the survey. Two measures of blood pressure were taken in a supine position after a 5 min rest; mean values were used. Participants were defined as having hypertension if they were treated for hypertension, or if their systolic/diastolic blood pressures were ≥140/90 mmHg. Weight and height were measured in lightly clad participants, and body mass index was calculated. The waist perimeter was measured horizontally at the smallest circumference between the lower rib and the iliac crests. Participants were followed until the end of the study or until a cardiovascular event occurred.

### Primary outcome

The primary outcome was the first occurrence of a myocardial infarction (fatal or not), a diagnosis of myocardial ischemia and/or significant coronary stenosis determined either by coronary angiogram or scintigraphy, revascularization therapy or a cerebral infarction. These events were physician adjudicated by medical record review with information provided by treating physicians and hospitals, in participants who, on the follow-up questionnaires, reported new cardiovascular disease, chest pain, stroke, or leg pain when walking.

### Biological Analyses

Blood was drawn after a 12-h fast. All biochemical measurements except bacterial DNA analysis were from one of four health center laboratories located in France at Blois, Chartres, La Riche, or Orléans. Fasting plasma glucose measured by the glucose oxidase method, was applied to fluoro-oxalated plasma using a Technicon RA100 (Bayer Diagnostics, Puteaux, France) or a Specific or a Delta device (Konelab, Evry, France). Total-cholesterol, HDL-cholesterol and triglycerides were measured by enzymatic methods; LDL-cholesterol was calculated from the Friedewald equation; fibrinogen was quantified by nephelometry and leucocytes by flow cytometry. Aspartate amino transferase (AST) and Alanine aminotransferase (ALT) activities were measured at 37°C with an automatic analyser (Technicon Dax 24; Bayer Diagnostics; or Lab 20 or Delta 60i; Konelab).

### Proteobacteria phylum quantification within blood microbiota

The 16S ribosomal DNA bacterial gene sequences were measured in blood collected at baseline. We studied the 16S rDNA gene as some regions of this gene are highly conserved between different species of bacteria and it is considered a marker of the overall microbiota [22]. In addition to highly conserved primer binding sites, the 16S rRNA gene sequences contain hypervariable regions that can provide species-specific signature sequences useful for bacterial identification. The 16S ribosomal DNA gene sequence common to the vast majority of the bacteria phyla (Eubac) and the 16S rDNA sequences that belong to the Proteobacteria phylum (Pbac) were measured in blood at baseline. The 16SrDNA qPCR technique has been established as follows. Using both sets of primers (Eubac and Pbac) the efficacy of PCR amplification has been calculated against a pure E. coliBl21 DNA standard curve from 0.001 to 10 ng/µl and found linear (∼95% efficacy). The rate of amplification was found close to 100%. It was calculated as well according to the following formula A = b.2^n^ where A is the expected amount of amplified DNA, b is the amount of DNA to be amplified, and n is the number of PCR cycles Similarly, the efficacy of PCR amplification for each sample has been established and found similar to that obtained for the standard curve. However, due to the large diversity of bacterial DNA in each sample, the rate of amplification could not be calculated precisely. This rate is expected to be better for Pbac PCR than for Eubac, based on the dominance of the Proteobacteriacae family [16]. Therefore, the ratios obtained between Pbac and Eubac are expected to be higher than 1.

### Bacterial DNA preparation

DNA was extracted from peripheral blood leukocytes using a classical phenol/chloroform extraction method followed by alcohol precipitation (ice-cold 70% alcohol). Air dried DNA was re-suspended in Tris EDTA and stored at −80°C before use [23], [24]. This method did not use glass microbeads to increase the efficiency of bacterial DNA extraction, which is commonly employed in current methods, as at the time of sample preparation, the isolation of bacterial DNA was not the goal of the work. However, despite this important technical difference, we succeeded in amplifying 16S rDNA from blood. To validate the difference between these two extraction procedures, we extracted total blood DNA in the presence and in the absence of microbeads. The efficacy of bacterial DNA extraction, as assessed by 16S rDNA qPCR amplification, was 10 times higher using microbead, although the absolute value of the bacterial DNA concentration was higher, the proportions between samples remained the same.

### Eubacteria quantitation

Total DNA concentration was determined using the Quant-iT^TM^ dsDNA Broad-Range Assay Kit (Invitrogen). The mean concentration was 121.1±208.3 ng/µl. Each sample was diluted ten-fold in Tris buffer EDTA. The DNA was amplified by realtime PCR (Stepone+; Applied Biosystems) in optical grade 96-well plates. The PCR reaction was performed in a total volume of 25 µl using the Power SYBR® Green PCR master mix (Applied Biosystems), containing 300 nM of each of the universal forward and reverse primers eubac-F (5′-TCCTACGGGAGGCAGCAGT-3′) and eubac-R (5′-GGACTACCAGGGTATCTAATCCTGTT-3′). The reaction conditions for amplification of DNA were 95°C for 10 min and 35 cycles of 95°C for 15 s and 60°C for 1 min. The amplification step was followed by a melting curve step according to the manufacturer's instructions (from 60°C to 90°C) to determine the specificity of the amplification product obtained. The amount of amplified DNA was determined using a standard curve obtained by real time PCR from dilutions ranging from 0.001 to 10 ng/µl of E. coli BL21 total DNA.

### Proteobacteria quantitation

The DNA coding the 16S rDNA was amplified as described above using forward and reverse primers Proteobac-F (F-Bact1369) (5′- CGGTGAATACGTTCCCGG-3′) and Proteobac -R (R-Prok1492) (5′- TACGGCTACCTTGTTACGACTT -3′) as described by Valladares et al [25]. These primers amplify a subset of 16SrDNA which corresponds mostly to Proteobacteria as determine by Blast analysis of the forward primer against the RDP database. The reaction conditions for amplification of DNA were 50°C for 2 min, 95°C for 10 min and 40 cycles of 95°C for 15 s, 60°C for 1 min and 72°C for 30 s. The amplification step was followed by a melting curve step according to the manufacturer's instructions (from 60°C to 95°C) to determine the specificity of the amplification product obtained. The amount of amplified DNA was determined using a standard curve obtained by real time PCR from dilutions ranging from 0.001 to 10 ng/µl of E. coli BL21 total DNA.

### Statistical analyses

SAS version 9.2 was used for statistical analysis. For analyses, the bacterial gene concentrations were log transformed, as the distributions were skewed; triglyceride concentrations were also log-transformed.

Baseline characteristics of participants are shown as mean ± SD, geometric */÷ e^SD^ (where SD is the standard deviation of the log-transformed variable) or as n (%), and they were compared between those destined to have acute cardiovascular events and those who were not, using t-tests or χ^2^ tests. Baseline characteristics of participants were compared over tertiles of Eubac and Pbac, by regression analysis for continuous variables and by the Armitage trend test for percentages. Standardised hazards ratios associated with cardiovascular events were calculated from Cox models, with age as the time variable, and after adjustment for sex, baseline hypertension, diabetes, smoking status, LDL-cholesterol, and fibrinogen. The proportional hazards assumption of the Cox model was checked by including an interaction term between the time variable and bacterial gene concentrations as continuous variables. The linearity of the relations with Eubac and Pbac were checked by the addition of a squared term to the models.

## Results

### Characteristics of the studied population

At baseline, among the 5212 participants in the D.E.S.I.R. study, 183 presented biological signs of infection or received antiviral therapy. The whole battery of major cardiovascular risk factors was not available in 77 volunteers, 490 did not undergo proteobacteria gene or Eubac gene determination and 526 were lost to follow-up. These volunteers were excluded from the analysis. They were significantly younger and less frequently smokers as compared with analyzed subjects. The characteristics of the study population (n = 3936) are shown in Table 1, and traditional risk factors were higher in those with incident cardiovascular events as compared with controls.

**Table 1:** Characteristics of the D.E.S.I.R. study population, mean ± SD, geometric */÷ e^SD^ or n(%), according to presence of incident cardiovascular events during the 9 year follow-up.

	Free of cardiovascular events (n = 3863)	With cardiovascular events (n = 73)	*P*
Men (n (%))	1895 (49)	65 (89)	<0.0001
Age (years)	47±10	55±8	<.0001
Body mass index (kg/m^2^)	24.6±3.7	26.8±3.7	<.0001
Waist perimeter (cm)	83±11	93±12	<.0001
Systolic blood pressure (mm Hg)	131±15	144±17	<.0001
Diastolic blood pressure (mm Hg)	80±9	86±10	<.0001
Total cholesterol (mmol/l)	5.71±0.97	6.21±1.07	<.0001
HDL-cholesterol (mmol/l)	1.64±0.43	1.38±0.37	<0.0001
LDL-cholesterol (mmol/l)	3.58±0.90	4.13±0.91	<.0001
Triglycerides (mmol/l) *	0.97*/÷1.67	1.35*/÷1.65	<.0001
Fasting blood glucose (mmol/l)	5.34±0.77	6.13±1.98	<.0001
Fibrinogen (g/l)	2.99±0.65	3.15±0.60	0.04
Leukocytes (10^9^/l)	6.36±1.71	7.14±1.95	0.0001
Aspartate aminotransferase (UI/l)	20.50±9.87	24.12±12.04	0.002
Alanine aminotransferase (UI/l)	25.63±16.88	32.75±21.07	0.0004
Eubac gene (ng/µl) * ‡, *	0.07*/÷2.86	0.05*/÷3.06	0.02
Pbac gene (ng/µl) * § *	0.20*/÷4.34	0.22*/÷5.18	0.7
Current smokers (n (%))	984 (25)	27 (37)	0.03
Hypertension (n (%))	1391 (36)	52 (71)	<0.0001
Diabetes (n (%))	84 (2)	13 (18)	<0.0001

* Log transformed for analysis.

‡ Eubac denotes 16S ribosomal DNA gene sequences, common to the vast majority of the bacteria phyla.

§ Pbac denotes 16S ribosomal DNA gene sequences that belongs to the Proteobacteria phylum.

**Figure 1:**
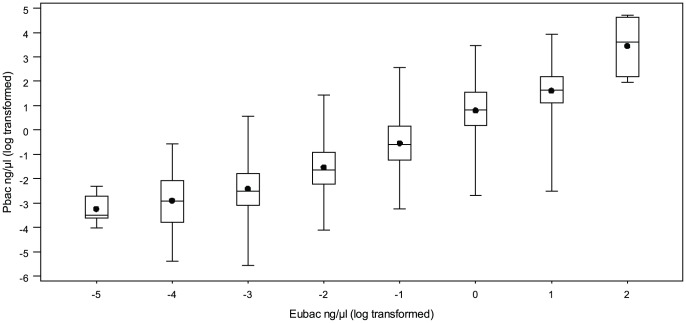
Proteobacteria phylum within blood microbiota. Box and wisker plot, showing medians (dot in middle of box, quartiles at end of box, and as wiskers the extremes), of Eucbacteria: 16S ribosomal DNA gene sequences common to the vast majority of bacteria phyla (Eubac) according to Pbac: 16S ribosomal DNA sequences that belong to the Proteobacteria phylum (both variables are log transformed).

Pbac was positively correlated with Eubac (r = 0.59; *P*<0.0001) (Figure 1). Similar relations were observed for Pbac and Eubac and major cardiovascular risk factors. Age, blood pressures, fibrinogen and ALT increased over tertiles of Pbac and Eubac, whereas the percentage of current smokers decreased (Tables 2, 3). No significant relation was reported with other traditional risk factors such as LDL-cholesterol or fasting blood glucose.

**Table 2:** Major cardiovascular risk factors by tertiles of Eubacteria (Eubac).

	Tertiles of Eubac			
	≤0.039 ng/µL	0.040 to 0.093 ng/µL	>0.093 ng/µL	*P trend*
	n = 1299	n = 1338	n = 1299	
Men (n (%))	638 (49)	652 (49)	670 (52)	0.1
Age (years)	46±10	47±10	48±10	0.0002
Body mass index (kg/m^2^)	24.7±3.7	24.6±3.7	24.6±3.5	0.5
Waist perimeter (cm)	83±12	83±12	83±11	0.6
Systolic blood pressure (mm Hg)	131±15	130±15	132±16	0.06
Diastolic blood pressure (mm Hg)	80±9	79±9	81±10	0.008
Total cholesterol (mmol/l)	5.74±0.98	5.73±0.97	5.71±0.97	0.3
HDL-cholesterol (mmol/l)	1.62±0.43	1.66±0.43	1.61±0.42	0.01
LDL-cholesterol (mmol/l)	3.60±0.91	3.58±0.90	3.58±0.90	0.3
Fasting blood glucose (mmol/l)	5.39±0.82	5.34±0.69	5.34±0.91	0.03
Fibrinogen (g/l)	2.93±0.58	2.95±0.60	3.12±0.74	0.0001
Aspartate aminotransferase (UI/l)	20.65±9.44	20.65±9.55	20.39±10.74	0.74
Alanine aminotransferase (UI/l)	25.19±15.55	25.43±17.56	26.67±17.74	0.06
Current smokers (n (%))	382 (29)	321 (24)	308 (24)	0.0004
Diabetes (n (%))	30 (2)	29 (2)	38 (3)	0.2

**Table 3:** Major cardiovascular risk factors by tertiles of Proteobacteria (Pbac).

	Tertiles of Pbac			
	≤0.095 ng/µL	>0.095 to <0.33	≥0.33 ng/µL	*P trend*
	n = 1299	n = 1338	n = 1299	
Men (n (%))	640 (49)	648 (48)	672 (52)	0.1
Age (years)	47±10	47±10	48±10	0.01
Body mass index (kg/m^2^)	24.8±3.8	24.5±3.6	24.7±3.7	0.3
Waist perimeter (cm)	83±12	83±11	84±12	0.8
Systolic blood pressure (mm Hg)	131±15	130±15	132±16	0.01
Diastolic blood pressure (mm Hg)	80±9	79±9	81±10	0.002
Total cholesterol (mmol/l)	5.74±0.98	5.73±0.97	5.71±0.97	0.4
HDL-cholesterol (mmol/l)	1.63±0.43	1.64±0.42	1.62±0.43	0.4
LDL-cholesterol (mmol/l)	3.60±0.91	3.58±0.90	3.58±0.90	0.5
Fasting blood glucose (mmol/l)	5.39±0.82	5.34±0.69	5.34±0.91	0.2
Fibrinogen (g/l)	2.93±0.58	2.95±0.60	3.12±0.74	0.0001
Aspartate aminotransferase (UI/l)	20.80±9.72	20.00±8.60	20.90±11.29	0.04
Alanine aminotransferase (UI/l)	25.55±15.85	24.22±14.60	27.56±19.96	<.0001
Current smokers (n (%))	362 (28)	353 (26)	296 (23)	0.001
Diabetes (n (%))	30 (2)	26 (2)	41 (3)	0.08

### Prediction of acute cardiovascular events and microbiota

**Figure 2:**
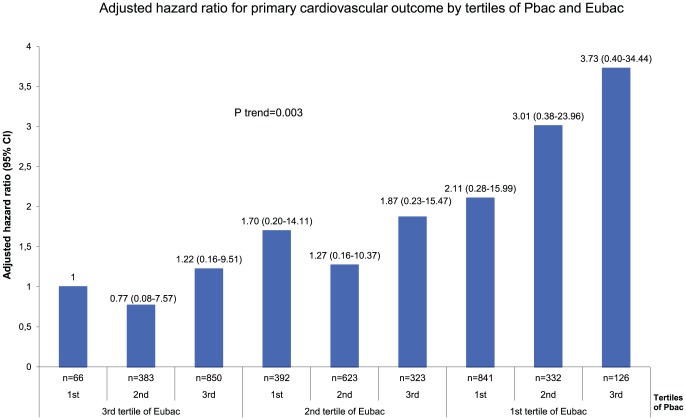
Adjusted Hazard ratio of having primary cardiovascular outcome by tertiles of Eubacteria (Eubac) and Proteobacteria (Pbac).

We recorded 73 incident cases of acute cardiovascular events, including 30 myocardial infarctions (2 fatal), 26 with revascularization therapy, 11 significant myocardial ischemia diagnosed either by coronarography or scintigraphy and 6 cranial infarctions during an average follow-up time of 101±22 months. Eubac was significantly lower in patients destined to have cardiovascular events. A non significant increase in Pbac concentration was also observed (Table 1). In multivariate Cox model analysis, after adjustment for confounders, Eubac was negatively correlated with primary outcome. When Pbac was added to the model, Eubac remained negatively correlated with the onset of primary outcome and Pbac was positively linked (Table 4). We present the hazard ratio for primary outcome by tertiles of Eubac and Pbac (Figure 2). A 3.7 fold increase in the risk of cardiovascular events was observed between volunteers who had the lowest levels of Eubac and the highest level of Pbac as compared with those who had the highest levels of Eubac and the lowest level of Pbac (*P* for trend 0.003).

**Table 4:** Standardized hazards ratios for an incident cardiovascular outcome.

	Hazard ratio(95% CI)	P value
Model 1		
Eubac	0.68 (0.53–0.88)	0.004
Model 2		
Eubac	0.50 (0.35–0.70)	<0.0001
Pbac	1.56 (1.12–2.15)	0.007

Models 1 and 2 were adjusted for gender, smoking status, LDL-cholesterol, fibrinogen, hypertension and diabetes. In addition , in model 2 Pbac was included.

## Discussion

The main finding of the study is that a dysbiosis in blood microbiota, defined by a decrease in blood bacterial DNA content and an increase in the proportion of Proteobacteria phylum within blood microbiota, predicts long-term cardiovascular prognosis. Since this study is the first to raise this concept, confirmatory study is required.

Until now, most of the epidemiological evidence that supports a relation between infection and cardiovascular events is based on associations between circulating antibacterial antibodies and cardiovascular diseases [7], [10]. For example, it has been shown that antibodies against oral pathogens were correlated either with target organ damage [26] or with the onset of cardiovascular events [27]. Based on this indirect approach, various pathogens were identified as predictors of cardiovascular diseases. Also, the presence of pathogens within plaque has been previously established [28], in coronary lesions as well: using human specimens obtained during coronary atherectomy, the “fingerprints” of more than 50 different bacterial species have been observed [29]. Therefore, it seems unlikely that a single pathogen is a key for the onset of cardiovascular events [30]. In addition to these results, our findings show that beyond the diversity of pathogens associated with cardiovascular diseases, tissue microbiota equilibrium could be involved in the mechanism underlying the relation between infection and atherosclerotic events.

The concept of microbiota dysbiosis as a key player in the course of various diseases has been proposed for gut [31]–[33] and oral microbiota [34], [35]. Changes in gut microbiota equilibria operate on pathological processes, either systematically through inflammatory mediators or locally. For example obesity has been associated with inflammation gut-mediated microbiota dysbiosis [36] whereas in inflammatory bowel disease, gut microbiota dysbiosis impacts on bile acid modification, which in turn has a direct deleterious effect on the disease [37]. To the best of our knowledge, our results are the first to show that microbiota dysbiosis is a player in pathological processes in cardiovascular disease.

This new paradigm may help to elucidate the relation between bacteria and cardiovascular disease. Indeed, whereas the aforementioned evidence supports the role of various pathogens, most interventional trials aiming to test antibiotics targeting these pathogens in the treatment of atherosclerosis, failed to improve cardiovascular prognostic [38]. In this respect, the resilience of the microbial community to a short-course antibiotic challenge has been repeatedly established [39], [40]. For example, in healthy volunteers the fecal microbiota showed a major shift in dominant species on antibiotic treatment, starting 24 h after treatment initiation. Within 30 days following antibiotic treatment, the fecal microbiota had an average similarity of 88% to baseline. It is likely that this resilience exists within human tissue and may contribute to explain the failure of antibiotic therapy administered over a short period of time, to prevent cardiovascular events. However, it seems possible to cause a shift to an alternative stable state. For example, the effect of repeated antibiotic perturbations have been tested in humans and it has been show that as soon as one week after the end of each course of antibiotics, gut microbiota begin to return to their initial state, but the return was often incomplete [41]. Taken together, these data may help to design some new interventional trials targeting tissue microbiota dysbiosis to prevent cardiovascular diseases.

Surprisingly, we observed a negative correlation between blood bacterial DNA as a whole or proteobacteria DNA and current smoking. In this respect, no change in lipopolysaccharide (LPS) concentration has been observed in an intervention trial [42] after cigarette smoking. Furthermore, no difference in blood LPS concentration has been found in current smokers as compared with non-smokers [43]. At this stage, no firm conclusion can be drawn from our current results. We can only hypothesize that an important change in tissue microbiota should be associated with current smoking. Importantly, we failed to find consistent relations between blood bacterial DNA and lipoprotein levels suggesting that these lipoproteins did not play a major role on blood bacterial DNA burden. Also, with respect to the body of evidence supporting a link between non-alcoholic fatty liver disease [44] and cardiovascular prognosis, we showed a positive correlation between Eubac and Pbac and ALT raising the interesting hypothesis of tissue microbiota dysbiosis as a common soil for non-alcoholic steatosis hepatitis and cardiac diseases.

In conclusion, our study demonstrates for the first time, that a microbiota dysbiosis predicts acute cardiovascular events in a large general population. This innovative concept opens new ways to predict and perhaps even prevent cardiovascular diseases.
